# Psychological Burden and Psychosocial Stress in Younger and Middle-Aged Women with Prior Myocardial Infarction

**DOI:** 10.3390/medicina62061082

**Published:** 2026-06-02

**Authors:** Mira Stipčević, Ivana Jurin, Marijana Knežević Praveček, Kristina Selthofer-Relatić, Laura Dražić, Marta Lugarić, Ognjen Čančarević, Šime Manola, Irzal Hadžibegović, Kristina Marić Bešić, Luka Matej Mahečić, Vedran Radonić, Ana Pavlović, Tomislav Krčmar, Matias Trbušić

**Affiliations:** 1 Department of Cardiology, General Hospital Zadar, 23000 Zadar, Croatia; mira.stipcevic@gmail.com; 2 Department of Cardiovascular Diseases, University Hospital Dubrava, 10000 Zagreb, Croatia; sime.manola@icloud.com (Š.M.); irzalh@gmail.com (I.H.); 3 Department of Cardiology, General Hospital Dr. Josip Benčević, 35000 Slavonski Brod, Croatia; marijana.pravecek@gmail.com; 4 Department of Cardiovascular Diseases, University Hospital Centre Osijek, 31000 Osijek, Croatia; selthofer.relatic@gmail.com; 5 Faculty of Medicine Osijek, Josip Juraj Strossmayer University of Osijek, 31000 Osijek, Croatia; 6 Department of Cardiology, University Hospital Centre Rijeka, 51000 Rijeka, Croatia; lori.drazic@gmail.com; 7 School of Medicine, University of Zagreb, 10000 Zagreb, Croatia; marta.lugaric03@gmail.com (M.L.); kmaricbesic@gmail.com (K.M.B.); matias.trbusic@gmail.com (M.T.); 8 Department of Cardiovascular Diseases, Sveti Duh Clinical Hospital, 10000 Zagreb, Croatia; ognjen200468@gmail.com; 9 Faculty of Dental Medicine and Health Osijek, Josip Juraj Strossmayer University of Osijek, 31000 Osijek, Croatia; 10 Department of Cardiovascular Diseases, University Hospital Centre Zagreb, 10000 Zagreb, Croatia; tomislav.krcmar@gmail.com; 11 Department of Cardiology, Merkur University Hospital, 10000 Zagreb, Croatia; lukamatejmahecic@hotmail.com (L.M.M.); vedran.radonic91@gmail.com (V.R.); 12 Zagreb-Centre Health Centre, 10000 Zagreb, Croatia; ana.bacekovic@gmail.com; 13 Faculty of Medicine, University of Rijeka, 51000 Rijeka, Croatia; 14 Department of Cardiology, University Hospital Centre Sestre Milosrdnice, 10000 Zagreb, Croatia

**Keywords:** myocardial infarction, women, psychological distress, psychosocial stress, depression, anxiety, cardiac rehabilitation

## Abstract

*Background and Objectives*: Younger and middle-aged women may continue to describe psychological and psychosocial burden years after myocardial infarction (MI), yet multicenter real-world data remain limited on what women retrospectively report during long-term follow-up. This study examined such reports in younger and middle-aged women with prior MI while explicitly accounting for delayed, heterogeneous follow-up timing and the non-standardized nature of symptom ascertainment. *Materials and Methods*: We performed a cross-sectional analysis within a Croatian multicenter cohort of 957 women younger than 60 years hospitalized with STEMI or NSTEMI between 1 February 2020 and 28 February 2025. Psychological information was collected retrospectively during routine follow-up; the formal PHQ-4 instrument was not administered. A study-specific four-item summary informed by PHQ-4 content was described, and exploratory multivariable analyses modelled this summary as a continuous variable. Descriptive timing-stratified sensitivity analyses and a timing-restricted continuous-score sensitivity model were additionally performed. *Results*: Prior psychiatric diagnosis was recorded in 16.0% of women, post-MI psychiatric diagnosis in 31.4%, and any psychosocial stressor in 73.2%. Among women with complete item responses, the four-item summary showed a broad distribution rather than a discrete threshold pattern. In continuous-score analyses, higher observed summary values were associated with younger age, prior psychiatric diagnosis, any psychosocial stressor, non-partnered status, non-employment, pregnancy complications/adverse pregnancy outcomes, and greater peri-menopausal symptom burden. Median summary values were only modestly higher in the small ≤ 1-year stratum and were otherwise similar across the later follow-up strata. Higher summary values were also associated with lower odds of self-reported regular current statin-based lipid-lowering use. *Conclusions*: These findings are best interpreted as exploratory data on retrospectively reported and currently endorsed long-term psychological and psychosocial burden years after MI, not as contemporaneous measures of recovery-phase psychopathology. Prospective studies with predefined assessment windows and validated instruments are needed.

## 1. Introduction

Myocardial infarction (MI) is increasingly recognized as both a biological event and a psychological disruption. Contemporary cardiovascular guidance no longer treats depression, anxiety, psychosocial stress, and trauma-related symptoms as peripheral to recovery; rather, these factors are relevant to adherence, functional recovery, participation in rehabilitation, and prognosis [1,2]. Across post-MI populations, pooled prevalence estimates remain clinically important, with depressive symptoms affecting roughly one quarter of survivors and anxiety or post-traumatic stress disorder (PTSD) affecting substantial minorities [3]. Meta-analytic work has likewise linked post-MI depression, anxiety, and acute coronary syndrome-related PTSD with recurrent events, mortality, and worse recovery trajectories [4,5,6,7].

From a psychosomatic and clinical-care perspective, the relevant burden after MI is broader than major depression alone. Recovery may be shaped by threat appraisal, fear of recurrence, altered illness perceptions, reduced perceived control, hypervigilance to bodily sensations, and disruption of identity and everyday roles [8,9,10,11]. These processes matter because they influence how women interpret symptoms, seek help, take medications, re-engage physically, and participate in rehabilitation or return-to-work trajectories [12,13,14,15,16,17,18].

This agenda is especially important for younger women. In the VIRGO programme and related studies, young women with MI reported more depressive symptoms, higher perceived stress, lower social support, more financial barriers, and less favorable health-status trajectories than similarly aged men [19,20,21,22,23,24]. More recent work has extended that signal to marital stress and broader women-centered stress biology, emphasizing that cardiovascular recovery in women is embedded in social roles, caregiving demands, and chronic psychosocial strain rather than infarct biology alone [25,26].

Women-focused and women-dominant MI phenotypes reinforce the same point. Among women with MI, psychosocial burden is prominent whether or not obstructive coronary disease is present, and survivors of spontaneous coronary artery dissection (SCAD) frequently describe PTSD, anxiety, depression, and resilience as central recovery constructs [27,28,29,30,31]. Qualitative studies likewise portray recovery as a prolonged negotiation of uncertainty, bodily vigilance, caregiving responsibilities, and personal meaning [32,33,34]. Yet multicenter real-world data remain limited for women younger than 60 years outside tightly protocolized acute psychocardiology studies. We therefore undertook a cross-sectional analysis within a multicenter cohort to describe retrospectively reported psychological symptoms, psychiatric history, and psychosocial stressors among younger women living with prior MI, and to explore how these observations might inform future prospective follow-up research.

## 2. Materials and Methods

### 2.1. Study Design and Setting

This report presents a cross-sectional analysis nested within a Croatian multicenter women-focused acute coronary syndrome (ACS) cohort conducted across participating cardiology centers. The source cohort was designed to capture clinical presentation, cardiovascular risk profile, women-specific history, psychiatric history, psychosocial context, treatment, and follow-up information in women hospitalized with ACS. For the present analysis, eligible index hospitalizations occurred from 1 February 2020 through 28 February 2025. The study is reported in accordance with the Strengthening the Reporting of Observational Studies in Epidemiology (STROBE) framework.

The analytic aim was descriptive and hypothesis-generating: to characterize retrospectively reported psychological burden after MI using the information available in the existing cohort. The analysis did not involve randomization, allocation to treatment, prospective mental-health intervention, or repeated standardized psychometric testing.

### 2.2. Study Population and Eligibility Criteria

The target population comprised women younger than 60 years at the time of the index hospitalization who had a final discharge diagnosis of MI, defined as ST-segment elevation myocardial infarction (STEMI) or non-ST-segment elevation myocardial infarction (NSTEMI). ACS subtype and MI phenotype were abstracted from hospital documentation and reflected the final diagnosis assigned by the treating cardiology team. Women hospitalized with unstable angina were excluded because the research question focused on recovery after infarction. All available eligible records meeting the hospitalization-period, age, and MI-subtype criteria were considered. Records were retained in descriptive analyses whenever the relevant variable was available. Participants with incomplete item-level mental-health data were excluded only from analyses that required construction of the four-item distress score or from complete-case regression models. After application of the eligibility criteria, the final study cohort comprised 957 women. No formal sample-size calculation was performed.

### 2.3. Data Sources and Follow-Up Assessment

Clinical data were obtained through retrospective review of routine hospital records and entered into predefined case-report fields at each participating center. Source documentation included discharge summaries, cardiology records, angiography or etiologic information when available, medication records, and structured cohort fields. Baseline variables included age, cardiovascular risk factors, ACS presentation, angiographic or etiologic MI phenotype, in-hospital clinical information, and women-specific history.

Post-discharge information was obtained during structured follow-up contact and from available routine follow-up records. The follow-up module captured psychiatric history, psychological symptoms reported during follow-up, psychosocial stressors, recorded advice or recommendation to seek psychiatric consultation, help-seeking, cardiac rehabilitation participation, physical activity, and medication-related variables. For lipid-lowering therapy, the cohort field asked whether the participant was taking the current lipid-lowering drug regularly at follow-up (code 1 = yes, 0 = no, 2 = not taking, 3 = using PCSK9/leqvio only). Accordingly, analyses of this field were interpreted as exploratory proxies for regular current statin-based lipid-lowering use rather than as formal adherence measures, and out-of-range values were treated as missing. When available, the date linked to the follow-up module was used to approximate the interval between the index MI and the follow-up contact contributing psychosocial information. Because this timing variable arose from routine follow-up documentation rather than a prescheduled psychometric visit, it should be interpreted as an approximation rather than as a standardized assessment time point.

Importantly, mental-health data were not collected through a protocol-mandated bedside psychocardiology assessment during the index hospitalization. Instead, women later described symptoms, feelings, stressors, and care experiences from memory during follow-up. The resulting variables therefore capture delayed, retrospectively reported, and currently endorsed burden during long-term follow-up rather than time-anchored symptoms from the acute or early post-MI period.

### 2.4. Mental-Health and Psychosocial Measures

The follow-up module recorded any psychiatric diagnosis before MI and any psychiatric diagnosis documented in follow-up records or reported by the participant after MI. Accordingly, these variables reflect routine clinical documentation and/or participant report rather than structured psychiatric ascertainment. Four symptom items, each scored from 0 to 3, covered two anxiety and two depressive symptom domains corresponding in content to those represented in the Patient Health Questionnaire-4 (PHQ-4) [35]. The formal PHQ-4 questionnaire, however, was not administered as a standardized instrument at a predefined post-MI visit. We therefore treated these items as a study-specific four-item symptom summary informed by PHQ-4 content, rather than as a formal PHQ-4 score or validated psychometric screen.

Among women with complete item-level responses, a four-item total summary (range, 0–12) was calculated descriptively. Because the items were recorded separately during routine follow-up rather than administered as the formal PHQ-4 questionnaire at a standardized time point, we do not interpret this total as a formal psychometric score. The main descriptive presentation therefore focuses on the observed summary distribution, reported in broad bands solely for readability. In the revised exploratory multivariable analyses, the four-item summary was modelled as a continuous study-specific outcome to avoid suggesting validated threshold-based screening categories.

Psychosocial stressors were recorded during follow-up and included job loss, divorce or separation, illness in the family, death of a close person, financial stress, work stress without job loss, and acute emotional stress. A composite variable for any psychosocial stressor was defined as endorsement of at least one of these domains.

### 2.5. Clinical, Social, and Women-Specific Variables

Clinical variables included age, ACS presentation (NSTEMI vs. STEMI), MI with non-obstructive coronary arteries (MINOCA), spontaneous coronary artery dissection (SCAD), hypertension, diabetes before hospitalization, dyslipidemia, and current smoking. MINOCA and SCAD were retained when documented by the treating center using angiographic, discharge, or etiologic information available in the medical record. Recovery-related secondary-prevention variables included participation in cardiac rehabilitation, self-reported regular physical exercise, and self-reported regular current statin-based lipid-lowering use at follow-up.

Social variables included partnership status and employment status at follow-up. Women-specific contextual variables included pregnancy complications or adverse pregnancy outcomes and peri-menopausal symptom burden. Pregnancy-related variables were derived from structured obstetric-history fields, including the item capturing stillbirth or preterm delivery. Peri-menopausal symptom severity was retained because menopausal transition, vasomotor symptoms, sleep disturbance, and mood symptoms may overlap during post-MI recovery and influence both distress reporting and care needs.

### 2.6. Data Handling and Statistical Analysis

Before analysis, variables were reviewed for range, internal consistency, missingness, and cross-center coding differences. Values outside the prespecified response range were treated as missing. Binary variables were harmonized to a common yes/no structure. Free-text psychiatric diagnoses were used only to support descriptive categorization when entries were interpretable; inferential analyses relied on structured variables to avoid overinterpreting heterogeneous narrative entries. No imputation was performed, and denominators are therefore reported explicitly as n/N. Information on cognitive status, memory performance, and contemporaneous standardized anxiety/depression assessments outside the four symptom items was not available in the source cohort and could not be included as covariates. Because the four-item symptom summary itself represented the only available current symptom measure, additional adjustment for independent contemporaneous depression/anxiety severity was not possible without circularity.

Continuous variables are summarized as median and interquartile range (IQR), because several variables, including the interval from hospitalization to the follow-up contact contributing psychosocial information, were not normally distributed. Categorical variables are summarized as counts and percentages using available-case denominators. The timing variable is reported to make clear that the psychological measures reflect heterogeneous routine follow-up rather than a standardized acute in-hospital or early post-MI assessment.

Exploratory multivariable analyses modelled the four-item summary as a continuous outcome using linear regression with HC3 robust standard errors. Covariates were selected a priori on clinical grounds and included age, ACS type (NSTEMI vs. STEMI), prior psychiatric diagnosis, any psychosocial stressor, partnership status, employment status, pregnancy complications/adverse pregnancy outcomes, and moderate/severe peri-menopausal symptoms. A separate multivariable logistic model examined whether higher continuous summary values were associated with self-reported regular current statin-based lipid-lowering use after adjustment for age, ACS type, prior psychiatric diagnosis, and any psychosocial stressor. Results are presented as beta coefficients or odds ratios (ORs) with 95% confidence intervals (CIs). Analyses were performed using complete-case data for variables included in each model. Statistical analyses were conducted in Python (version 3.11), and two-sided *p* values below 0.05 were considered statistically significant.

All statistical models should be interpreted as hypothesis-generating. The study was not designed to establish causal effects, diagnose psychiatric disorders, or evaluate symptom trajectories over time. As additional sensitivity analyses requested during peer review, we stratified participants descriptively by the interval from index hospitalization to the follow-up contact contributing psychosocial information (≤1 year, 1–3 years, and >3 years) and repeated the continuous-score multivariable model among women contacted within ≤3 years of MI. Because the earliest stratum was small, because follow-up timing remained heterogeneous even within these bands, and because symptom reporting was still retrospective rather than obtained at a predefined psychometric visit, these analyses were interpreted only as sensitivity analyses and were not used to infer temporal comparability or symptom trajectories.

### 2.7. Ethical Considerations

The parent multicenter study was conducted in accordance with the Declaration of Helsinki and was approved through local ethics procedures at the contributing clinical institutions covered by the available approval documentation. Available approval documentation includes the Ethics Committee of General Hospital Dr. Josip Benčević, Slavonski Brod (14 October 2025; receipt/approval no. 040000/25-72), University Hospital Centre Sestre milosrdnice (Class 003-06/25-03/055; Ref. 251-29-11/3-10; 13 October 2025), University Hospital Dubrava (approval no. 2025/1204-8; 4 December 2025), University Hospital Centre Zagreb (Class 8.1-25/313-4; No. 02/013 AG; 8 December 2025), and Merkur University Hospital (Ref. 03/I-1867; 3 March 2026). A subsequent written clarification from the Ethics Committee of University Hospital Dubrava (approval no. 2025/1204-8; 6 March 2026) confirmed that the protocol was reviewed as a retrospective, non-interventional study based on routinely collected pseudonymized/anonymized data and that individual informed consent was not required for the conduct of the research. The approved procedures comprised retrospective extraction of routine clinical information and structured follow-up contact under the approved protocol. Data were collected and analyzed in pseudonymized form, and access to protected study records was restricted to authorized investigators.

## 3. Results

### 3.1. Participant Characteristics and Timing of Follow-Up Assessment

The study cohort included 957 women younger than 60 years with MI whose index hospitalizations occurred from 1 February 2020 through 28 February 2025. Median age was 53 (IQR, 48–57) years. STEMI was present in 541/957 women (56.5%) and NSTEMI in 416/957 (43.5%). MINOCA was recorded in 123/957 women (12.9%) and SCAD in 46/955 (4.8%). Hypertension was present in 556/957 (58.1%), diabetes before hospitalization in 132/957 (13.8%), dyslipidemia in 537/957 (56.1%), and current smoking in 605/950 (63.7%) (Table 1).

The timing of psychosocial assessment was heterogeneous and often remote from the index event. Among women with valid non-negative dates (n = 939), the interval from hospitalization to the follow-up contact contributing psychosocial information, or to the corresponding documented follow-up encounter, was 1268 (IQR, 766–1628) days, underscoring that the psychological information reflects delayed retrospective reporting rather than a standardized contemporaneous assessment during the acute admission. Within these 939 women, 44 (4.7%) were assessed within ≤1 year, 346 (36.8%) between 1 and 3 years, and 549 (58.5%) after >3 years; 18 women had missing or implausible interval data and were therefore excluded from the time-stratified sensitivity summary.

### 3.2. Retrospectively Reported Psychological Symptoms and Psychosocial Stressors

When women were contacted during long-term follow-up, many retrospectively described psychological symptoms and psychosocial stressors that remained salient in how they now understood and remembered life after MI. A prior psychiatric diagnosis was recorded in 151/946 women (16.0%), and a psychiatric diagnosis during follow-up after MI in 286/911 (31.4%). Among women with complete four-item data, observed summary values spanned the full 0–12 range (Table 2), indicating heterogeneity rather than a discrete threshold pattern. At least one psychosocial stressor was reported by 666/910 women (73.2%) (Table 2).

Among women with a recorded prior psychiatric diagnosis and interpretable free-text entries, depressive, anxiety-related, and trauma- or adjustment-related diagnoses predominated.

At least one psychosocial stressor was reported by 666/910 women (73.2%). The most frequent stressors were acute emotional stress in 357/909 (39.3%), work stress in 236/908 (26.0%), illness in the family in 181/909 (19.9%), financial stress in 172/907 (19.0%), and death of a close person in 157/909 (17.3%). A recorded recommendation or advice to seek psychiatric consultation was documented in 180/890 women (20.2%), whereas 307/903 (34.0%) reported that they had sought help.

In descriptive time-stratified sensitivity analyses, the overall qualitative pattern of symptom burden and psychosocial stress remained visible across all three follow-up intervals, although the earliest stratum was small. Among women with complete four-item data, median summary values were 4.0 (IQR, 2.2–6.5) within ≤1 year, 3.0 (1.0–5.0) at 1–3 years, and 3.0 (1.0–6.0) after >3 years. Any psychosocial stressor was reported by 73.5%, 69.9%, and 75.5% of women with available data in these strata, respectively (Supplementary Table S2). In an additional timing-restricted continuous-score sensitivity model among women contacted within ≤3 years (n = 320), the broad direction of association for age, prior psychiatric diagnosis, psychosocial stress, partnership status, employment status, and peri-menopausal symptom burden remained similar, although precision was lower and pregnancy-related variables were attenuated (Supplementary Table S3). Because only 44 women fell within the ≤1-year stratum and only 18 had complete four-item data, these results remain sensitivity analyses and do not re-establish a standardized recovery-phase frame.

### 3.3. Exploratory Multivariable Associations with the Study-Specific Four-Item Summary

In multivariable analysis using the four-item summary as a continuous outcome, higher observed summary values clustered with prior psychiatric diagnosis and markers of social vulnerability. Younger age within this already young cohort was associated with higher summary values (beta −0.06 per year, 95% CI −0.09 to −0.03), while prior psychiatric diagnosis (beta 2.54, 95% CI 2.06 to 3.01), any psychosocial stressor (beta 0.53, 95% CI 0.14 to 0.92), non-partnered status (beta 0.50, 95% CI 0.13 to 0.86), non-employment (beta 0.57, 95% CI 0.21 to 0.93), any pregnancy complication/adverse pregnancy outcome (beta 0.40, 95% CI 0.06 to 0.75), and greater peri-menopausal symptom burden (beta 0.58, 95% CI 0.24 to 0.92) were associated with higher observed summary values (Table 3). NSTEMI was not independently associated with the summary score.

In a second multivariable model, each one-point increase in the four-item summary was associated with lower odds of self-reported regular current statin-based lipid-lowering use after adjustment for age, acute coronary syndrome type, prior psychiatric diagnosis, and any psychosocial stressor (OR 0.88, 95% CI 0.83 to 0.94; complete-case n = 752) (Table 3).

## 4. Discussion

### 4.1. Principal Findings

This cross-sectional analysis within a multicenter women-focused cohort should be read as an analysis of long-term, retrospectively reported psychological and psychosocial burden among women living with prior MI, not as a measurement study of recovery-phase psychopathology. When younger and middle-aged women were contacted years later, many described symptoms and stressors that remained salient in how they now understood, remembered, and lived with the event. The most defensible object of inference is therefore not early post-MI distress, but long-term retrospectively reported and currently endorsed burden among women living years after MI.

The social patterning of symptom burden was one of the clearest findings. Higher observed summary values clustered with prior psychiatric diagnosis, psychosocial stress, non-partnered status, and non-employment, suggesting that emotional burden after MI is carried within a wider matrix of vulnerability. This is consistent with work linking worse recovery in young adults to low social support, financial barriers, marital stress, and partner status [21,22,25,36], and with broader women’s cardiovascular literature emphasizing the role of chronic psychosocial strain and gendered social roles in shaping cardiovascular health [26]. Notably, this pattern was observed when the four-item summary was analysed as a continuous variable rather than through a pseudo-screening threshold, and the direction of association remained similar in the ≤3-year sensitivity subset, although precision was lower.

In this cohort, the median interval between the index MI and the follow-up contact contributing psychosocial information exceeded 3 years. At such intervals, the dataset no longer approximates post-MI distress close to the index event. Instead, it reflects what women later recalled, reinterpreted, and endorsed after subsequent symptoms, medical encounters, life events, treatment experiences, and adaptation [37,38,39].

This distinction is not merely semantic. Memory after a major cardiovascular event is reconstructive rather than archival. Repeated medical encounters, ongoing symptoms, family and work disruptions, recurrent cardiovascular concerns, and present affect may reshape how the infarction and its aftermath are remembered. Mood-congruent recall may amplify negative recollection when current distress is higher, whereas adaptation may compress or soften earlier burden. Consequently, the study cannot distinguish persistent symptoms from recurrent, newly developed, or later-contextualized symptoms, and some responses may reflect long-term illness narratives, present-day burden, and the psychology of living with prior MI as much as they reflect the original post-MI period.

A descriptive sensitivity analysis stratified by follow-up interval (≤1 year, 1–3 years, and >3 years) did not remove this interpretive problem. Median summary values were modestly higher in the small ≤1-year stratum and otherwise similar across later strata, while psychosocial stressors remained frequent throughout. Likewise, a timing-restricted continuous-score sensitivity model among women contacted within ≤3 years showed broadly similar directions of association but did not recover a true recovery-phase cohort or solve the underlying problem of retrospective ascertainment. In other words, simple banding of routine follow-up dates cannot recover a true recovery-phase design; it only shows that remembered and currently endorsed burden is not confined to one narrow segment of the available follow-up window.

### 4.2. External Context and Directions for Prospective Research

Our data do not justify implementation claims, screening strategies, or structured service models. The more defensible implication is narrower: women living with prior MI may continue to describe psychological and psychosocial burden years after the index event, and this long-term burden merits prospective study with predefined assessment windows and validated instruments [40,41,42,43,44].

Broader multidisciplinary or women-centered psychocardiology models are therefore referenced here only as external background concepts from prior literature, not as conclusions derived from this cohort. Their plausibility comes from prior work on social roles, rehabilitation, MINOCA and SCAD, and post-ACS follow-up, whereas the present dataset can only suggest that long-term burden and psychosocial vulnerability remain relevant topics for future prospective evaluation. The most informative next step would be prospective work using predefined assessment windows and repeated validated measures, or, in datasets where dates and sample sizes allow, sensitivity analyses restricted to shorter follow-up intervals.

The same caution applies to specialized cardiovascular nursing. In heart failure care, nurse-specialist consultations have been associated with more effective medication titration and improved cardiovascular outcomes, suggesting that structured nurse-led follow-up may be worth testing in other cardiovascular settings; however, our data do not evaluate such models and they are cited only to contextualize future research directions [40].

Accordingly, Supplementary Table S1 is presented only as a literature-informed outline of questions, domains, and possible assessment windows for future prospective research. It is not a study-derived pathway, validated model, or implementation proposal.

The present dataset is more directly informative about how long-term burden may co-travel with markers of vulnerability than about service design. Psychological burden may intersect with medication use, rehabilitation attendance, physical activity, return to work, smoking, and health-care use [16,17,18,42,45,46,47]. In our data, higher continuous summary values were associated with lower odds of self-reported regular current statin-based lipid-lowering use after adjustment, but this should be interpreted only as cross-sectional co-occurrence within delayed follow-up data, not as evidence that symptom burden causally impaired medication behavior.

Intervention literature is therefore relevant only as background for future trials. Collaborative care, structured psychological interventions, rehabilitation-based metacognitive therapy, internet-delivered cognitive-behavioral approaches, mindfulness-based programmes, and women-focused cardiac rehabilitation have all been studied in related settings [48,49,50,51,52,53,54,55,56,57,58], but the present dataset neither tests these approaches nor indicates which model should be implemented.

Thus, the principal contribution of the present study is to show that long-term psychological and psychosocial burden remains a salient part of the lived experience of many women with prior MI. It does not estimate formal psychiatric prevalence after MI, assess recovery-phase psychopathology, or justify service-design recommendations. The added descriptive timing-stratified sensitivity analysis and the timing-restricted continuous-score sensitivity model likewise do not restore temporal comparability; they simply show that the broad pattern of remembered and currently endorsed burden is not confined to one narrow segment of the available follow-up window.

### 4.3. Strengths and Limitations

These limitations are balanced by several strengths. The study draws on a sizeable multicenter cohort focused specifically on women with MI, captures psychiatric history, symptom burden, and psychosocial stressors within the same dataset, and situates these findings within a women-specific clinical context, including MINOCA and SCAD. Such material is uncommon in MI research and is valuable precisely because it reflects the realities of later follow-up outside narrowly protocolized acute-phase trials. For that reason, the present analysis is best viewed as hypothesis-generating evidence that can inform prospective studies using validated instruments at predefined time points.

This study has several limitations. It was a cross-sectional analysis nested within an existing multicenter cohort and not a psychocardiology study designed prospectively around standardized mental-health end points. Most importantly, women were not systematically screened during the index hospitalization or at a predefined post-MI time point. Instead, psychological information was obtained at heterogeneous routine follow-up contacts occurring a median of more than 3 years after MI. Accordingly, the data reflect delayed retrospective reporting and current endorsement rather than contemporaneous early post-MI assessment. This creates substantial temporal ambiguity and makes it impossible to determine whether endorsed symptoms were persistent from the index event, recurrent after a prior remission, newly developed later, or mainly reconstructed through subsequent experiences and present affect. The cohort also did not include dedicated measures of cognitive status, memory function, or concurrent mood specifically designed to disentangle recall processes.

The source cohort did not include a comprehensive, internally consistent psychometric battery; rather, it contained selected psychiatric-history variables, four symptom items aligned with PHQ-4 content, and psychosocial stressor fields collected in routine follow-up. The formal PHQ-4 questionnaire was not administered, and the derived four-item summary should therefore be interpreted as a pragmatic cohort-specific signal of retrospectively recalled and currently endorsed psychological burden rather than as a validated screening score. In this revision, the main multivariable analyses were deliberately expressed through continuous-score modelling to reduce threshold-based overinterpretation; nonetheless, even continuous modelling cannot overcome the retrospective nature of ascertainment. Psychiatric diagnoses after MI were based on routine documentation and/or participant report rather than structured adjudication. Missing data, variable denominators, and the absence of a male comparator or older female comparator further limit inference.

## 5. Conclusions

Among younger and middle-aged women living years after MI, psychological symptoms and psychosocial stressors were frequently described when participants were contacted long after the index event. Because assessment was delayed, heterogeneous, and retrospective, these findings are best interpreted as exploratory data on remembered and currently endorsed long-term psychological and psychosocial burden rather than measures of acute or early post-MI psychopathology. They cannot distinguish persistent from recurrent or newly developed symptoms and do not support direct conclusions about screening models or service design. Descriptive timing-stratified and timing-restricted sensitivity analyses did not eliminate these limitations. Their main value is to motivate prospective studies using validated instruments at predefined post-MI intervals and, where possible, repeated within-person measurements.

## Figures and Tables

**Table 1:** Baseline Clinical, Social, and Follow-Up Characteristics of the Study Cohort.

Characteristic	Value
**Index MI presentation**	
Age, years	53 (48–57)
STEMI	541/957 (56.5%)
NSTEMI	416/957 (43.5%)
MINOCA	123/957 (12.9%)
SCAD	46/955 (4.8%)
**Baseline cardiovascular profile**	
Hypertension	556/957 (58.1%)
Diabetes before hospitalization	132/957 (13.8%)
Dyslipidemia	537/957 (56.1%)
Current smoker at baseline	605/950 (63.7%)
**Social and women-specific context**	
Not partnered	239/916 (26.1%)
Not employed	308/900 (34.2%)
Any pregnancy complication/adverse pregnancy outcome	243/957 (25.4%)
Moderate/severe peri-menopausal symptoms	432/908 (47.6%)
**Recovery and follow-up context**	
Cardiac rehabilitation attendance	290/861 (33.7%)
Regular exercise	376/869 (43.3%)
Self-reported regular current statin-based lipid-lowering use	579/779 (74.3%)
Time from index hospitalization to psychosocial follow-up contact, days	1268 (766–1628)

Values are n/N (%) or median (IQR). Denominators vary because of missing data. MINOCA, myocardial infarction with non-obstructive coronary arteries; SCAD, spontaneous coronary artery dissection.

**Table 2:** Psychiatric History, Study-Specific Four-Item Summary, and Psychosocial Stressors During Follow-Up.

Variable	Value
**Psychiatric history**	
Any prior psychiatric diagnosis	151/946 (16.0%)
Any post-MI psychiatric diagnosis documented or reported at follow-up	286/911 (31.4%)
Study-specific four-item summary	
Observed summary-value band: 0–2	386/897 (43.0%)
Observed summary-value band: 3–5	284/897 (31.7%)
Observed summary-value band: 6–8	191/897 (21.3%)
Observed summary-value band: 9–12	36/897 (4.0%)
**Psychosocial stressors**	
Any psychosocial stressor	666/910 (73.2%)
Acute emotional stress	357/909 (39.3%)
Work stress without job loss	236/908 (26.0%)
Illness in the family	181/909 (19.9%)
Financial stress	172/907 (19.0%)
Death of a close person	157/909 (17.3%)
Divorce/separation	72/908 (7.9%)
Job loss	40/905 (4.4%)
Mental-health follow-up actions	
Recorded recommendation/advice to seek psychiatric consultation	180/890 (20.2%)
Help sought during follow-up	307/903 (34.0%)

Values are n/N (%). The bands shown below are descriptive display bins of the observed four-item total-summary distribution and do not correspond to validated thresholds, severity categories, or diagnostic boundaries. The formal PHQ-4 instrument was not administered. Out-of-range values were treated as missing.

**Table 3:** Exploratory Multivariable Associations with the Study-Specific Four-Item Summary and with Regular Current Statin-Based Lipid-Lowering Use.

Variable	Adjusted Estimate	95% CI	*p* Value
**Panel A: Continuous four-item summary as outcome (beta coefficients)**			
Age, per year	−0.06	−0.09 to −0.03	<0.001
NSTEMI (vs. STEMI)	−0.13	−0.47 to 0.20	0.436
Prior psychiatric diagnosis	2.54	2.06 to 3.01	<0.001
Any psychosocial stressor	0.53	0.14 to 0.92	0.008
Not partnered	0.50	0.13 to 0.86	0.008
Not employed	0.57	0.21 to 0.93	0.002
Any pregnancy complication/adverse pregnancy outcome	0.40	0.06 to 0.75	0.023
Moderate/severe peri-menopausal symptoms	0.58	0.24 to 0.92	<0.001
**Panel B: Regular current statin-based lipid-lowering use (odds ratios)**			
Four-item summary, per 1-point increase	0.88	0.83 to 0.94	<0.001
Age, per year	1.01	0.99 to 1.04	0.373
NSTEMI (vs. STEMI)	0.89	0.64 to 1.24	0.501
Prior psychiatric diagnosis	1.05	0.66 to 1.66	0.848
Any psychosocial stressor	1.04	0.71 to 1.51	0.857

Panel A shows robust linear regression with the continuous four-item summary as the outcome (n = 848). Panel B shows logistic regression for regular current statin-based lipid-lowering use (n = 752). The formal PHQ-4 instrument was not administered; the four-item summary is a study-specific descriptive summary informed by PHQ-4 content. In Panel B, regular current statin-based use was defined from the follow-up field as code 1 = yes versus codes 0/2/3 not meeting that definition; out-of-range values were treated as missing. Beta, unstandardized regression coefficient; OR, odds ratio; CI, confidence interval.

## Data Availability

The data are not publicly available because they derive from a multicenter clinical cohort containing sensitive patient-level information. De-identified data may be made available by the corresponding author on reasonable request and with permission of the contributing clinical centers, subject to applicable ethics and data-protection requirements.

## Supplementary Materials

The following supporting information can be downloaded at: https://www.mdpi.com/article/10.3390/medicina62061082/s1, Table S1, literature-informed domains and possible assessment windows for future prospective research after myocardial infarction in women; Table S2, descriptive sensitivity analysis stratified by the interval from MI to psychosocial follow-up contact; and a STROBE checklist for observational cohort reporting; Table S3, Sensitivity Analyses Using Continuous Modelling of the Study-Specific Four-Item Summary.
