# Supplementary material for: Psychological Burden and Psychosocial Stress in Younger and Middle-Aged Women with Prior Myocardial Infarction
Source: Medicina (Kaunas). 2026 Jun 2;62(6):1082. doi: 10.3390/medicina62061082 (PMC13303980; doi:10.3390/medicina62061082)
Supplement: Supplementary file 1 [file medicina-62-01082-s001.zip › medicina-4300451-supplementary.pdf]

Supplementary Materials

Supplementary Table S1. Literature-Informed Domains and Possible Assessment Windows for Future Prospective Research in Women With Prior Myocardial Infarction

This table provides external contextual considerations for future prospective studies. It is not derived from the present cohort and should not be interpreted as a recommended care pathway, implementation model, or service-design proposal.

Potential assessment window in future prospective studies	Examples of validated constructs that could be assessed	Potential disciplines that future prospective studies could involve
After clinical stabilization / before discharge (future studies only)	Brief validated symptom screening; major stressors, social support, perceived control, and functional red flags	Cardiology clinician, cardiac nurse, and where indicated mental-health input as part of prospective study design
Early outpatient follow-up or rehabilitation entry (future studies only)	Repeated validated symptom measures; sleep, function, rehabilitation participation, return-to-work concerns, and medication use	Outpatient cardiology, rehabilitation, psychology/psychiatry, and social-work disciplines depending on study aims
Longer-term follow-up at predefined intervals (future studies only)	Repeated symptom measures, evolving stressors, work status, help-seeking, and symptom persistence or recurrence	Cardiology, primary care, and mental-health disciplines according to prospective protocol
Problem-triggered or recurrent-event reassessment in future studies	Validated reassessment when symptoms recur, new stressors emerge, medication use worsens, or new cardiovascular events occur	Study-specific re-entry pathways defined prospectively rather than inferred from the present cohort

Supplementary Materials

Supplementary Table S2. Descriptive Sensitivity Analysis Stratified by the Interval from Myocardial Infarction to the Follow-Up Contact Contributing Psychosocial Information

This descriptive table was added in response to peer review to show how the observed four-item summary and key psychosocial variables were distributed across broad timing strata. These bands do not restore temporal comparability, because symptom reporting remained retrospective and the earliest stratum was small.

Interval from MI to follow-up contact	Women in stratum, n	Complete four-item data, n	Four-item summary median (IQR)	Summary 0–2	Summary 3–5	Summary 6–12	Any psychosocial stressor	Sought help
≤1 year	44	18	4.0 (2.2–6.5)	5/18 (27.8%)	8/18 (44.4%)	5/18 (27.8%)	25/34 (73.5%)	7/19 (36.8%)
1–3 years	346	333	3.0 (1.0–5.0)	160/333 (48.0%)	102/333 (30.6%)	71/333 (21.3%)	235/336 (69.9%)	105/338 (31.1%)
>3 years	549	541	3.0 (1.0–6.0)	218/541 (40.3%)	173/541 (32.0%)	150/541 (27.7%)	403/534 (75.5%)	194/541 (35.9%)

Values are n/N (%) unless otherwise indicated. Summary bands are descriptive displays of the observed study-specific four-item total-summary distribution and should not be interpreted as validated screening categories or diagnostic thresholds. Denominators vary because of missing data.

Supplementary Materials

Supplementary Table S3. Sensitivity Analyses Using Continuous Modelling of the Study-Specific Four-Item Summary

Panel A shows a timing-restricted continuous-score sensitivity model among women contacted within <=3 years of the index MI. Panel B shows the association between the continuous four-item summary and self-reported regular current statin-based lipid-lowering use. The formal PHQ-4 instrument was not administered; the four-item summary is a study-specific descriptive summary informed by PHQ-4 content.

Variable	Adjusted estimate	95% CI	p value
**A. Continuous four-item summary among women contacted within <=3 years (beta coefficients)**			
Age, per year	-0.05	-0.10 to -0.01	0.026
NSTEMI (vs. STEMI)	-0.44	-0.96 to 0.09	0.102
Prior psychiatric diagnosis	2.43	1.56 to 3.30	<0.001
Any psychosocial stressor	0.66	0.05 to 1.27	0.034
Not partnered	0.96	0.37 to 1.54	0.001
Not employed	0.96	0.37 to 1.56	0.002
**Any pregnancy complication / adverse pregnancy outcome**	**0.20**	**-0.35 to 0.76**	**0.472**
Moderate/severe peri-menopausal symptoms	0.69	0.17 to 1.22	0.010
B. Regular current statin-based lipid-lowering use (odds ratios)			
Four-item summary, per 1-point increase	0.88	0.83 to 0.94	<0.001
Age, per year	1.01	0.99 to 1.04	0.373
NSTEMI (vs. STEMI)	0.89	0.64 to 1.24	0.501
Prior psychiatric diagnosis	1.05	0.66 to 1.66	0.848
Any psychosocial stressor	1.04	0.71 to 1.51	0.857

Panel A: HC3-robust linear regression, n = 320. Panel B: logistic regression, n = 752. In Panel B, regular current statin-based use was defined from the follow-up field as code 1 = yes versus codes 0/2/3 not meeting that definition; out-of-range values were treated as missing.
